# Using Chemistry To Recreate the Complexity of the Extracellular Matrix: Guidelines for Supramolecular Hydrogel–Cell Interactions

**DOI:** 10.1021/jacs.4c02980

**Published:** 2024-06-18

**Authors:** Laura Rijns, Matthew B. Baker, Patricia Y. W. Dankers

**Affiliations:** † Institute for Complex Molecular Systems (ICMS), Eindhoven University of Technology, 5600 MB Eindhoven, The Netherlands; ‡ Department of Biomedical Engineering, Laboratory of Chemical Biology, Eindhoven University of Technology, 5600 MB Eindhoven, The Netherlands; § Department of Complex Tissue Regeneration, MERLN Institute for Technology Inspired Regenerative Medicine, Maastricht University, 6200 MD Maastricht, The Netherlands; ∥ Department of Instructive Biomaterials Engineering, MERLN Institute for Technology Inspired Regenerative Medicine, Maastricht University, 6200 MD Maastricht, The Netherlands; ⊥ Department of Chemical Engineering and Chemistry, Eindhoven University of Technology, 5600 MB Eindhoven, The Netherlands

## Abstract

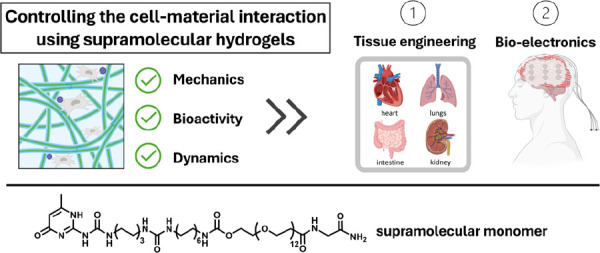

Hydrogels have emerged as a promising class of extracellular matrix (ECM)-mimicking materials in regenerative medicine. Here, we briefly describe current state-of-the-art of ECM-mimicking hydrogels, ranging from natural to hybrid to completely synthetic versions, giving the prelude to the importance of supramolecular interactions to make true ECM mimics. The potential of supramolecular interactions to create ECM mimics for cell culture is illustrated through a focus on two different supramolecular hydrogel systems, both developed in our laboratories. We use some recent, significant findings to present important design principles underlying the cell–material interaction. To achieve cell spreading, we propose that slow molecular dynamics (monomer exchange within fibers) is crucial to ensure the robust incorporation of cell adhesion ligands within supramolecular fibers. Slow bulk dynamics (stress–relaxation—fiber rearrangements, τ_1/2_ ≈ 1000 s) is required to achieve cell spreading in soft gels (<1 kPa), while gel stiffness overrules dynamics in stiffer gels. Importantly, this resonates with the findings of others which specialize in different material types: cell spreading is impaired in case substrate relaxation occurs faster than clutch binding and focal adhesion lifetime. We conclude with discussing considerations and limitations of the supramolecular approach as well as provide a forward thinking perspective to further understand supramolecular hydrogel–cell interactions. Future work may utilize the presented guidelines underlying cell–material interactions to not only arrive at the next generation of ECM-mimicking hydrogels but also advance other fields, such as bioelectronics, opening up new opportunities for innovative applications.

## Introduction

One of the grand challenges of chemistry is to observe nature in its complexity yet try to recreate, or dare to go beyond, with simplicity. For example, the extracellular matrix (ECM) is a complex system of biomacromolecules that provides an environment for and regulates cellular behavior. Mimicking the structural, mechanical, biochemical, and dynamic properties of the ECM stands to benefit many biomedical applications. Recreating the ECM is a grand challenge that calls upon chemists to unravel the secrets of nature and reconstruct its complexities in a reductionist, simplistic manner. Yet, this is far from straightforward. Nature, with elegance, showcases the remarkable capabilities of molecular design via the ECM. But, the daunting task of a chemist lies in deciphering its macromolecular blueprint and translating this into synthetic and controllable frameworks. In this exploration of design parameters for creating ECM mimics, we should facilitate conversations between chemistry and biology: materials and cells. In this perspective we showcase how, via chemical design, we are revealing key features important to the molecular construction of synthetic ECMs from supramolecular components.

### Native ECM and Its Three Important Properties

**Figure 1:**
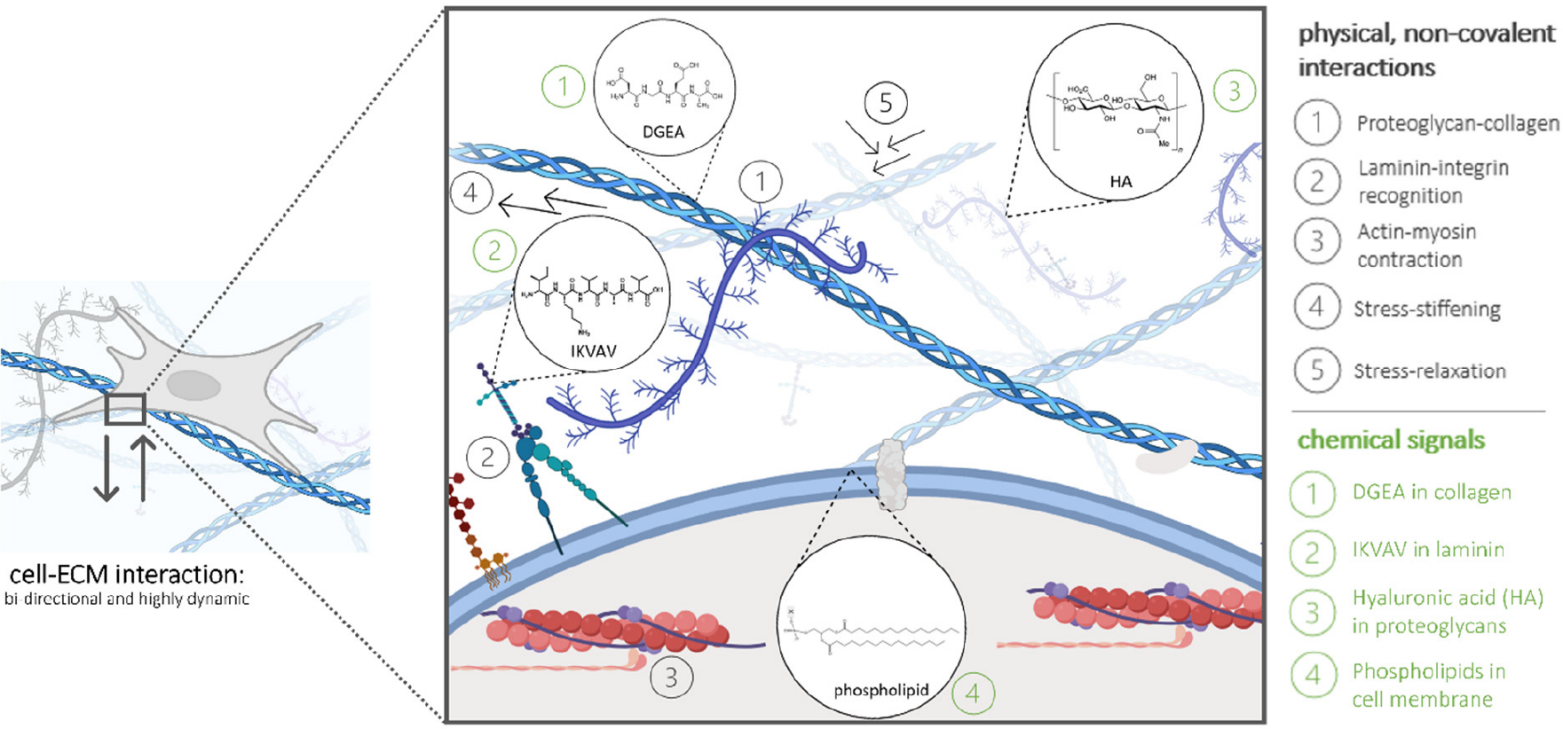
Cell–ECM interactions are bidirectional and highly dynamic. The ECM makes physical, noncovalent interactions with cells (gray) and offers chemical signals to cells (green). Figure created using Biorender software.

The ECM is a multicomponent, dynamic network in which cells reside. Recreating this physical, dynamic, and (bio)chemical environment to interface with living systems is important to many fields—from regenerative medicine to wearable electronics.^1−5^ The ECM regulates cell differentiation, proliferation, and fate and enables the effective homeostasis and communication within living tissues. The ECM is composed of two main classes of macromolecules: proteoglycans, formed from glycosaminoglycans (GAGs), and fibrous proteins, for example, collagen. Nature uses these two important types of natural building blocks to build the ECM.^6,7^ This complex combination creates a responsive network with both elastic and viscous properties,^8^ which provides both chemical signals to cells and engages in physical, noncovalent interactions with cells (Figure 1).^9,10^

**Figure 2:**
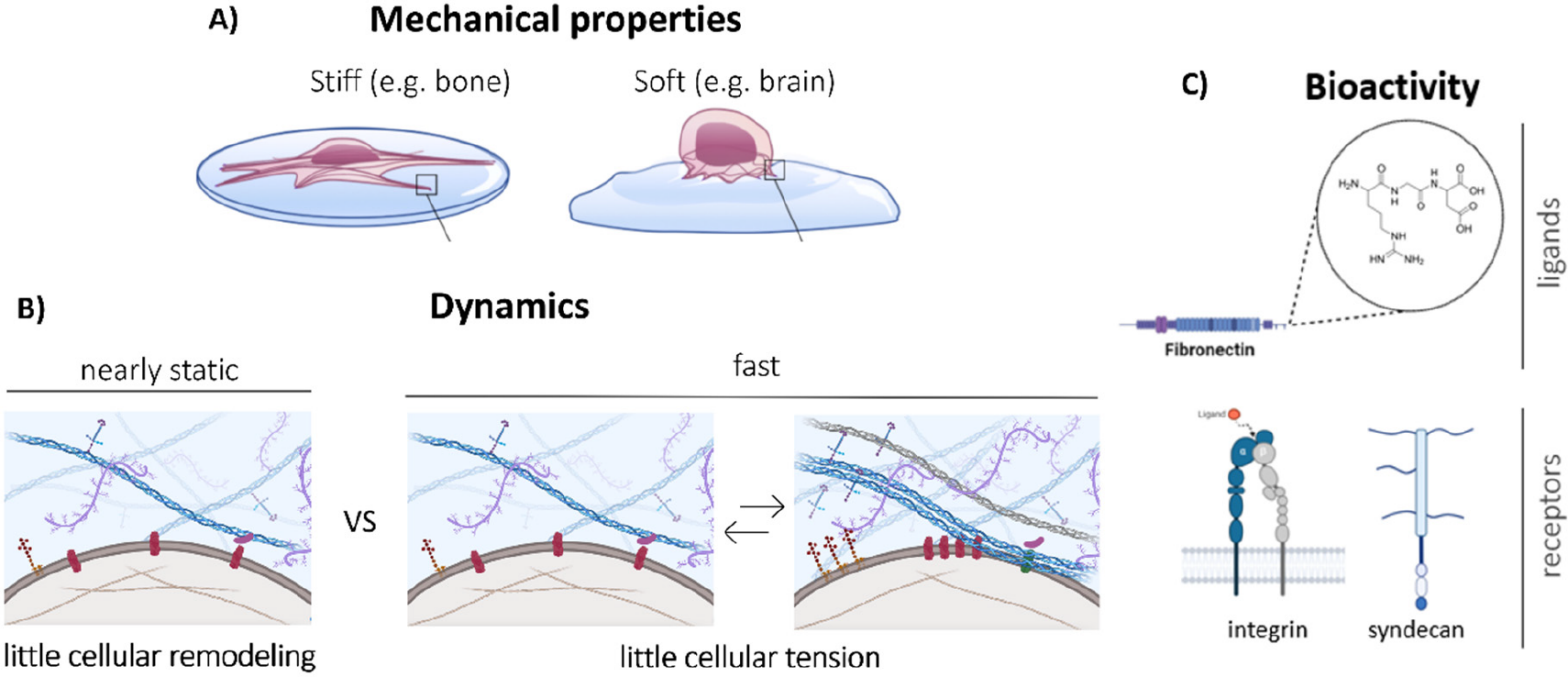
Overview of important properties of the ECM influencing cellular behavior, classified as the (A) mechanical, (B) dynamic, and (C) bioactive properties. Adapted with permission from ref (27). Copyright 2021 Springer Nature.

When recreating this ECM, it is essential to capture its essential characteristics: the mechanical properties,^11^ bioactivity,^12,13^ and dynamics^14^ (Figure 2). For the mechanical properties, the concentration, variety, and hierarchical order of proteins can differ greatly between tissues, leading to variations in tissue stiffnesses, ranging from 1.9 kPa in the lungs^15^ to 20 GPa in cortical bone.^16^ Additionally, specific relaxation mechanisms and an increase in stiffness upon an applied stress may occur, i.e., stress stiffening, which also impact cellular behavior.^17,18^ Next to the macroscopic bulk stiffness of tissues, it is important to realize that the ECM itself is a highly dynamic, multicomponent network, primarily held together by noncovalent interactions.^19^ Proteoglycans and glycoproteins can both store and dissipate deformation energy, displaying a time-dependent mechanical response, which results in stress–relaxation behavior when being deformed, or creep when a mechanical stress is applied.^20^ Next to the ECM’s complex mechanical and dynamic properties, the biochemical information of the ECM also greatly impacts cellular behavior. An abundant and important receptor that regulates many ECM–cell interactions is the integrin receptor.^21,22^ Integrins interact with the extracellular world by binding to ECM glycoproteins, like laminin, collagen, and fibronectin, and transmit this information into the cytoplasm of a cell, thereby regulating many cell–ECM interactions.^23^ Additionally, the above-mentioned properties often interfere with each other, as time-dependent behavior for both the mechanical and the biochemical side of the ECM is often observed. Aside from the mechanical, bioactive, and dynamic features of the ECM, other properties influence cellular behavior, like structural and topographic properties as well as fiber type, diameter, and orientation.^24−26^

### Cell–Material Communication

The ECM has a reciprocal interaction with the cell. The cell influences and remodels the matrix, and the matrix provides information to steer cellular behavior (Figure 1). This two-way communication provides a highly dynamic, responsive, and remodeling relation between a living unit (the cell) and secreted macromolecules (the ECM). Whether we look at the stress–relaxation, the reorganization of a matrix, degradation, or presentation of latent growth factors, the reliance on supramolecular interactions is crucial to many functions within the cellular environment. Recreation of the environment around cells is extremely important; it can lead to the success or failure of a clinical strategy or a medical device. Thus, we must understand how this cell–material communication operates in order to design and synthesize functional and successful biomaterials that can interface with living systems. Importantly, these dynamic interactions create time scales within the cellular environment for various processes, and matching material time scales is imperative.^28^

### Current State-of-the-Art Hydrogels as ECM Mimics

ECM-mimicking materials that communicate with living systems can guide cell behavior—from healing tissue to immunomodulation. An important class of biomaterials is hydrogels, having undergone a boom in rational design.^29,30^ The highly hydrated, macromolecular, and porous structure of hydrogels favor nutrient and oxygen transport needed for cell growth and proliferation.^31^ Hydrogels based on natural materials (e.g., Matrigel, collagen^32^) offer great biological activity but have poor reproducibility and sometimes a tough path to clinical translation. Hydrogels based on synthetic materials offer great control, tailorability, and scalability, yet their chemical simplicity makes advanced mechanical properties and bioactivity challenging to achieve. Hybrid materials, synthetic in nature but chemically modified, can at times balance the best features and minimize the drawbacks of each strategy but remain difficult to control.

**Figure 3:**
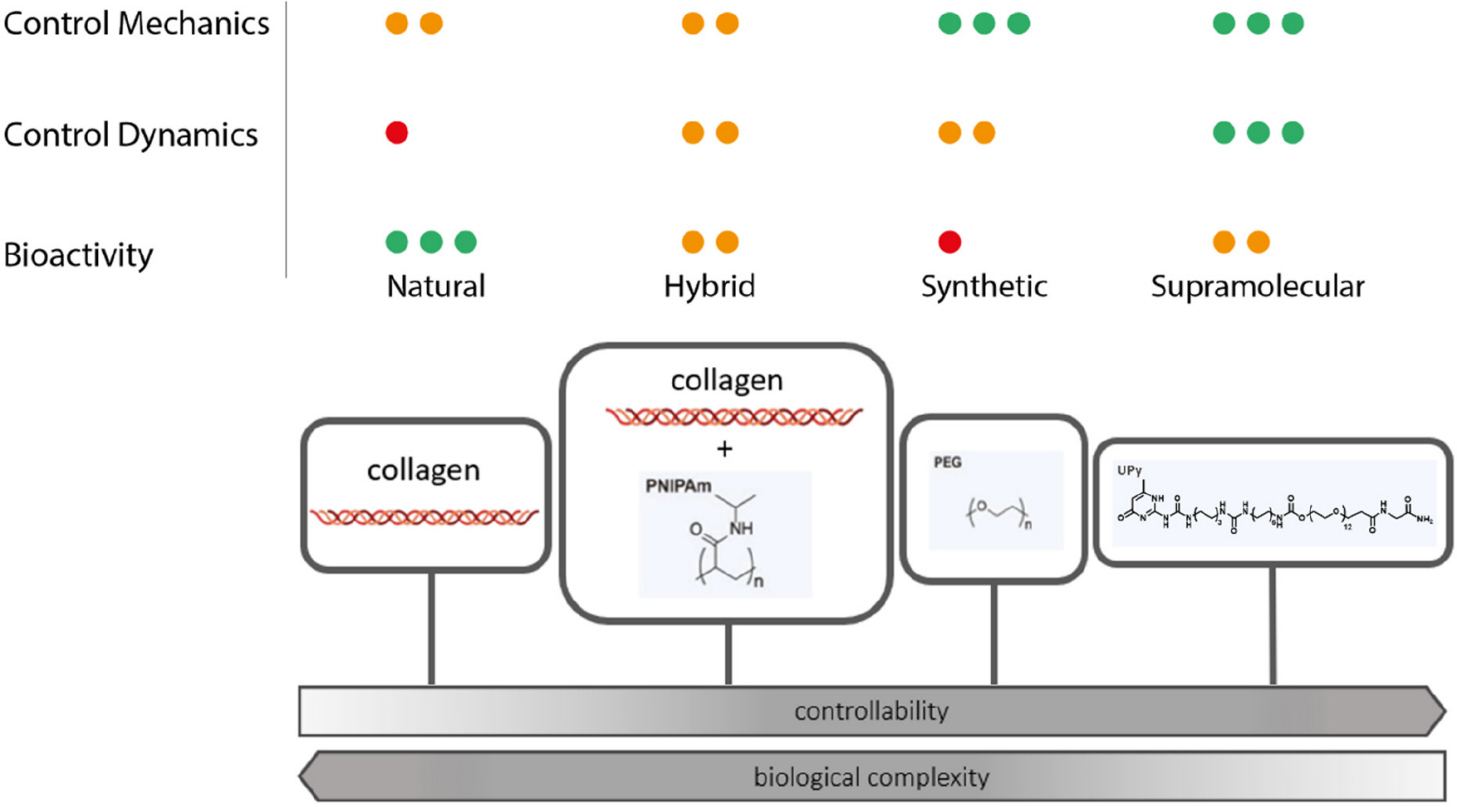
Overview of current state-of-the-art ECM mimics, classified as natural, hybrid, synthetic covalent, and (synthetic) supramolecular, ranked by their biological complexity and controllability, and rated for their ability to control and provide mechanical, dynamic, and bioactive properties toward cells, with the green dot representing excellence, the orange dot mediocrity, and the red dot unsatisfactory performance.

Traditionally, hydrogel design strategies have revolved around static, covalently cross-linked hydrogels and offer mechanical tunability based on well-defined structure–property relationships based off of decades of study and theory (Figure 3). Complex mechanical features, like stress stiffening by polyisocyanide (PIC) polymers (i.e., a stiffening of the material upon an applied force), were successfully introduced into this class of covalent hydrogels.^33^ Although the irreversible nature of the cross-links brings elastic properties to hydrogels, these materials generally lack the dynamic nature of the ECM that enables tissue regeneration and integration.^34^ Therefore, cross-links based on physical, noncovalent or reversible, covalent chemistries^35^ have gained increasing interest as they impart dynamic, viscoelastic properties into hydrogels (Figure 3). Furthermore, these strategies can also introduce biomimetic hierarchical structure into hydrogels. For example, the diameter (thickness) of covalent polymers is typically a few Angstroms versus a few nanometers for supramolecular fibers;^36,37^ for comparison, native ECM fibers have a diameter of several tens of nanometers (∼77 nm)^38^ - which will influence cellular behavior.^39,40^ Such dynamic hydrogels, held together by reversible cross-links between their macromolecules, further add desirable properties for biomedical applications such as self-healing, stress–relaxation,^41^ and stress stiffening.^42^ Yet, these materials require careful design and optimization to match the viscoelastic time scales of the material to the time scales of cellular processes.^28^ Recently, advances in supramolecular chemistry^43−45^ and the creation of hybrid and multicomponent hydrogels are providing a more simple path toward recreating the complexity and dynamics found in natural systems (Figure 3). Supramolecular hydrogels merge the unique advantages of hydrogels and supramolecular chemistry, bringing self-healing, on-demand reversible gelation, stress–relaxation, and stimuli responsiveness, among other interesting properties.^46,47^ These hydrogels can be designed using a variety of reversible noncovalent cross-links, including π–π interactions, hydrogen bonds, host–guest interactions, metal coordination, and hydrophobic and electrostatic interactions, offering customized properties to match a diverse range of native tissues.

Mimicking and recreating suitable biological environments is a critical endeavor. From regenerative medicine to advanced soft matter, drug delivery, medical devices, and wearables, the ability to interface with and control biological tissue unlocks the potential in many future applications. In this perspective, we propose a supramolecular approach to recapitulate the complexity of the ECM in a simplistic manner using self-assembling monomers to fabricate larger, hierarchical hydrogels with complex mechanical, bioactive, and dynamic features. The supramolecular design and properties of other supramolecular materials are first discussed. We then use some recent, significant findings to present some emerging design principles underlying supramolecular hydrogel–cell interactions. This perspective ends with discussing some considerations of the supramolecular approach as well as provides future prospects to further improve control over supramolecular hydrogel–cell interactions.

## Simplicity Enables Complexity; A Supramolecular Approach

Looking at the native ECM, many of the components are quite simple in their makeup (e.g., proteins built from amino acids), yet the complexity emerges from their precise combinations, stereochemical complexity, tertiary structure, and supramolecular interactions. As we move toward the recreation of nature’s complex matrix, we must strive to use simple and minimal components, which via smart combinations can be leveraged to create complex function.

Supramolecular interactions are reversible, noncovalent interactions which can give rise to supramolecular assemblies/aggregates (nondirectional interactions, like van der Waals forces) or more well-defined supramolecular polymers (directional interactions, like hydrogen bonding). Supramolecular interactions are arising as a very promising tool to create ECM mimics owing to their inherent dynamics, adaptability, and tunability. Bioactive function can easily be introduced through coupling bioactive cues to the monomeric building blocks and mixing these into the supramolecular assemblies.^48−50^ Supramolecular copolymerization can elegantly be used to tune not only the ligand presentation^41^ but also the fiber morphology^51^ and mechanical properties.^41,52^ Varying the formulation of these molecules allows the formation of fibers, hydrogels, and solid meshes as well as the tuning of hydrogel properties.^53,54^ Finally, the coassembly of individual systems into larger, hierarchical complexes with synergistic function might be key to recapitulate all important properties of the native ECM.

**Figure 4:**
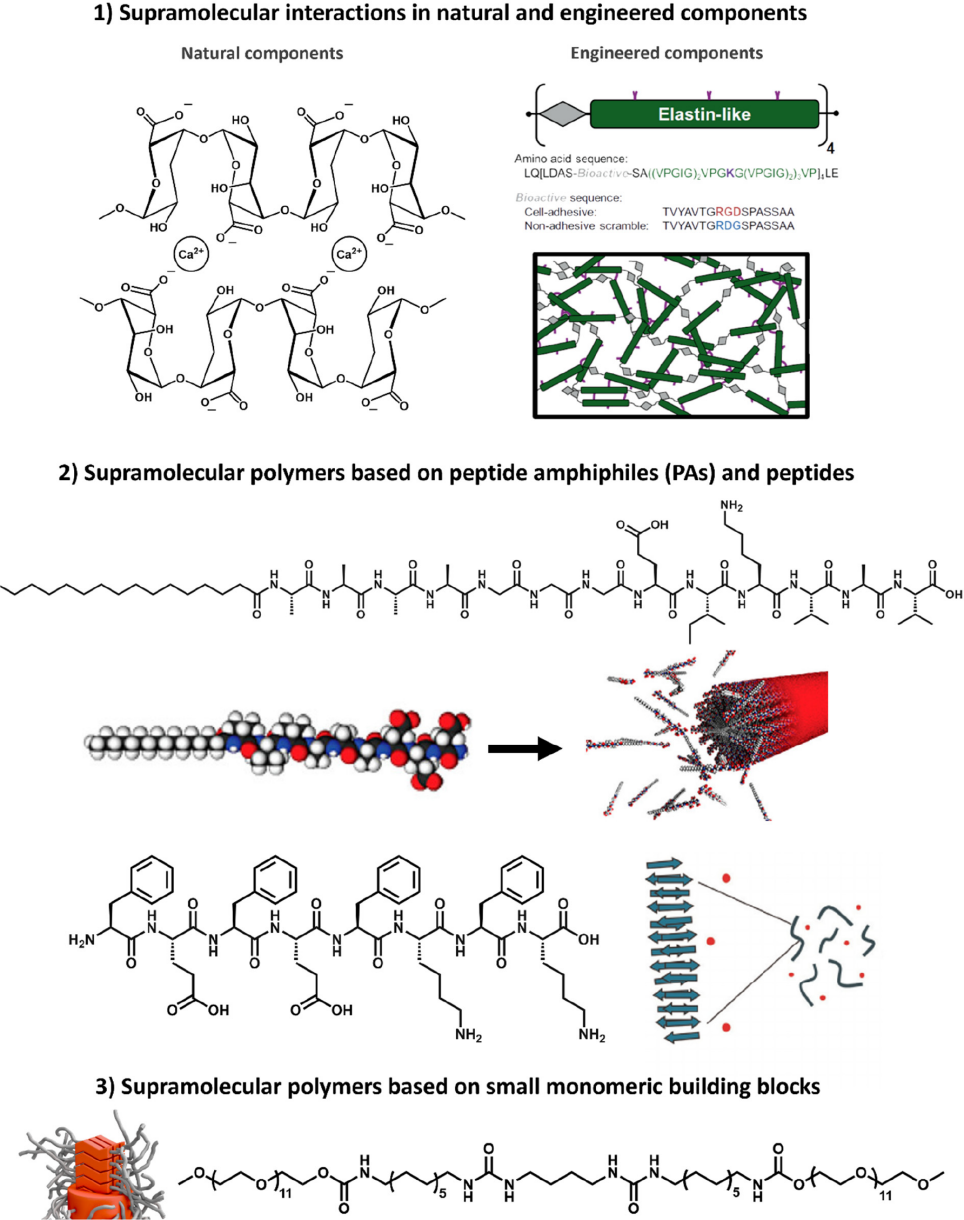
Overview of different synthetic biomaterials based on supramolecular interactions, classified as follows. (1) Supramolecular interactions in natural and engineered components. Adapted with permission from ref (67). Copyright 2013 Elsevier. (2) Supramolecular polymers based on peptide amphiphiles (PAs) and peptides. Adapted with permission from ref (55). Available under a CC-BY 4.0. Copyright 2017 ACS. Adapted with permission from ref (56). Available under a CC-BY 3.0. Copyright 2014 Elsevier. (3) Supramolecular polymers based on small monomeric building blocks.

We here discuss promising classes of biomaterials based on supramolecular interactions, classified, for ease of discussion, as (1) supramolecular interactions in natural and engineered components, (2) supramolecular polymers based on peptide amphiphiles (PAs) and peptides, and (3) supramolecular polymers based on small monomeric building blocks (Figure 4).

### Supramolecular Interactions Based on Natural and Engineered Components

One way to design hydrogels is based on long polymer chains, which can be composed of natural or engineered components (Figure 4-1). Alginate, for example, is a linear polysaccharide consisting of mannuronic acid and guluronic acid. Alginate can easily be obtained, has limited toxicity, and forms gels conveniently via the use of divalent cations (e.g., calcium or barium).^57^ By covalently coupling PEG to alginate, Chaudhuri et al. proposed perturbation of calcium cross-linking and formed hydrogels with similar mechanical stiffness but varying stress–relaxation and creep.^14,58^ High stress–relaxation enhanced cell spreading owing to the cells remodeling their substrate. In addition, control over the mechanical and dynamic properties is introduced by using different concentrations of calcium together with different molecular weight alginate.^14^ Alginate is often used to impart processability and ideal to control the dynamic mechanical properties. However, alginate is itself not biodegradable in the human body, and alginate hydrogels erode unpredictably over time due to ion exchange of calcium with surrounding media, causing chains to dissolute.^59^ In addition, cell adhesion to alginate is limited and requires functionalization for protein binding and attachment of cells.^60,61^

Examples of hybrid hydrogels include bovine serum albumin (BSA) networks reinforced with noncovalently adsorbed polyelectrolytes, as studied by Khoury et al.^62^ Besides the reinforcement and large stiffening effect, the noncovalently attached polyelectrolytes can create and break local bonds, allowing the gels to heal any structural damage and function as a shape memory material.^62^ Koenderink et al. showed the importance of an increase in complexity by combining two natural ECM components, i.e., collagen and hyaluronic acid (HA).^63^ The interaction of HA inside and around collagen fibers created a soft hydrated matrix, interacting and stabilizing collagen. Additionally, the presence of HA shifted the stress-stiffening response, lowering the sensitivity but increasing the stability, as the stress needed to break the network increased.^63^ Azevedo et al. used positively charged, amphipathic peptides for the supramolecular cross-linking of native hyaluronan and could regulate the structural and mechanical properties of the resulting hybrid hydrogels through the peptide sequence employed.^64^ Overall, such hybrid gels offer great potential to combine different properties (i.e., one labile and one slow degrading gel) within a single material.^65^

To introduce structural organization in biomaterials, elastin-like proteins (ELPs), short repetitive sequences which provide elasticity and mechanical stability, can be used and have been well studied by the Heilshorn lab.^66,67^ Due to the nature of these ELPs, their exact mechanical properties can be tuned via changes in the amino acids or by the assembly conditions.^68^ By tuning the elastic sequences and by utilizing RGD as a ligand, materials with specific cellular adhesion signals can be created.^67^ Another strategy includes the use of recombinant proteins to fabricate hydrogels, pioneered by the Tirrell lab.^69,70^ The incorporation of noncanonical amino acids here is very appealing as it allows chemical modification of a specific region of interest in the protein. Overall, the modularity and biocompatibility of protein-based biomaterials makes them good candidates to investigate fundamental cell–matrix processes.

### Supramolecular Polymers Based on Peptide Amphiphiles (PAs) and Peptides

Moreover, supramolecular interactions in and between peptide amphiphiles (PAs) and peptides can be used to form supramolecular polymers (Figure 4-2). PAs are designed by functionalizing amphiphilic building blocks with a peptide sequence. A good amphiphilic building block consists of a hydrophobic domain to shield water, a hydrogen bonding region, and a polar headgroup.^71^ Additionally, a charged region could be introduced to allow responsiveness upon applying external stimuli, such as pH^72^ and salt concentration,^73,74^ or functional units could be attached for cellular targeting.^74,75^ By varying the design of these specific regions, differently shaped amphiphiles have been created that assemble into different large structures.^76^ PAs are often based on the interaction between hydrophobic and hydrophilic interactions, e.g., IKVAV, which is also a bioactive moiety (Figure 4-2).^77^ This PA is based on hydrophilic sequences with hydrophobic tails of glycine and the alkyl moieties, which align upon self-assembly into fibers that entangle into a polymeric network.^73^ Variations in the hydrophilic and hydrophobic domains allow for control over the structural as well as mechanical properties of the hydrogel.^78^ The most well-known PA hydrogel that forms fibrous structures is the commercial PuraMatrix, based on RADA sequences.^79^ These sequences form stable β-sheets through electrostatic forces and hydrophobic interactions. Finally, the Mata lab made great contributions in understanding multicomponent, PA-based supramolecular hydrogels for regenerative medicine applications.^80^

Variations on these PAs include self-assembling peptide hydrogels (SAPHs) and other short peptide sequences, such as the RADA sequence.^81^ A well-known sequence in SAPHs is FEFEFKFK, created by the Saiani lab, which self-assembles into antiparallel β sheet nanofibers upon increasing the pH or ionic strength of the solution and forms self-supporting gels above the critical gelation concentration.^82^ The Adams, Ulijn, Xu, and Tuttle groups made great efforts on the screening of various combinations of short tripeptides in their ability to self-assemble.^80,83−87^ The Collier and Pochan laboratories have beautifully crafted SAPHs by ingeniously leveraging longer peptide fragments, either drawing inspiration from the intricate designs found in nature’s own proteins (Collier lab)^88^ or focusing specifically on block polypeptides (Pochan lab) to induce hydrogel formation.^89^ Recent reports on the modulation of dynamics within PA structures have shown the powerful potential of gaining control over this elusive property.^90−92^

### Supramolecular Polymers Based on Small Monomeric Building Blocks

A last class of supramolecular biomaterials includes the assembly of monomeric building blocks to form fiber-like structures (Figure 4-3). In this class, bola-amphiphiles are often used, where a hydrophobic core is shielded by two hydrophilic end groups (Figure 4-3), increasing the solubility and allowing the design of various shapes and structures.^93^ The Sijbesma and Palmans groups designed materials based on complementary bis-urea motifs,^94−96^ separated via an aliphatic spacer and shielded with OEG blocks, yielding well-defined micellar rods in aqueous solutions (Figure 4-3). Assemblies could easily be tuned by varying the length of PEG and the size of the aliphatic linker, thereby tuning the mechanical properties and yield strain (Figure 4-3).^97^ Additionally, by incorporating azide- and ethyne-functionalized bis-urea compounds, covalent cross-links could be introduced between the self-assembled rods,^98^ surprisingly exhibiting stress-stiffening behavior (Figure 4-3). This property was attributed to soft bending modes, present in bundles of bis-urea fibers, leading to this unusual stress response.^98^ The modular nature of the supramolecular system also allowed easy incorporation of bioactive moieties by conjugating short peptide sequences (now as the bioactive ligand and not as a self-assembling moiety) to a urea motif and mixing it into the material.^99^ So, overall, supramolecular hydrogels formed by the noncovalent assembly between monomers provide great and independent control over the hydrogel’s mechanical, bioactive, and dynamic properties.

Other supramolecular monomers include the benzene-1,3,5-tricarboxamide (BTA)^100^ and ureido-pyrimidinone (UPy) motif,^101^ both developed in our laboratories. They are both able to form supramolecular fibers and hydrogels and are discussed in full detail in the next section.

#### BTA-Based Supramolecular Hydrogels

**Figure 5:**
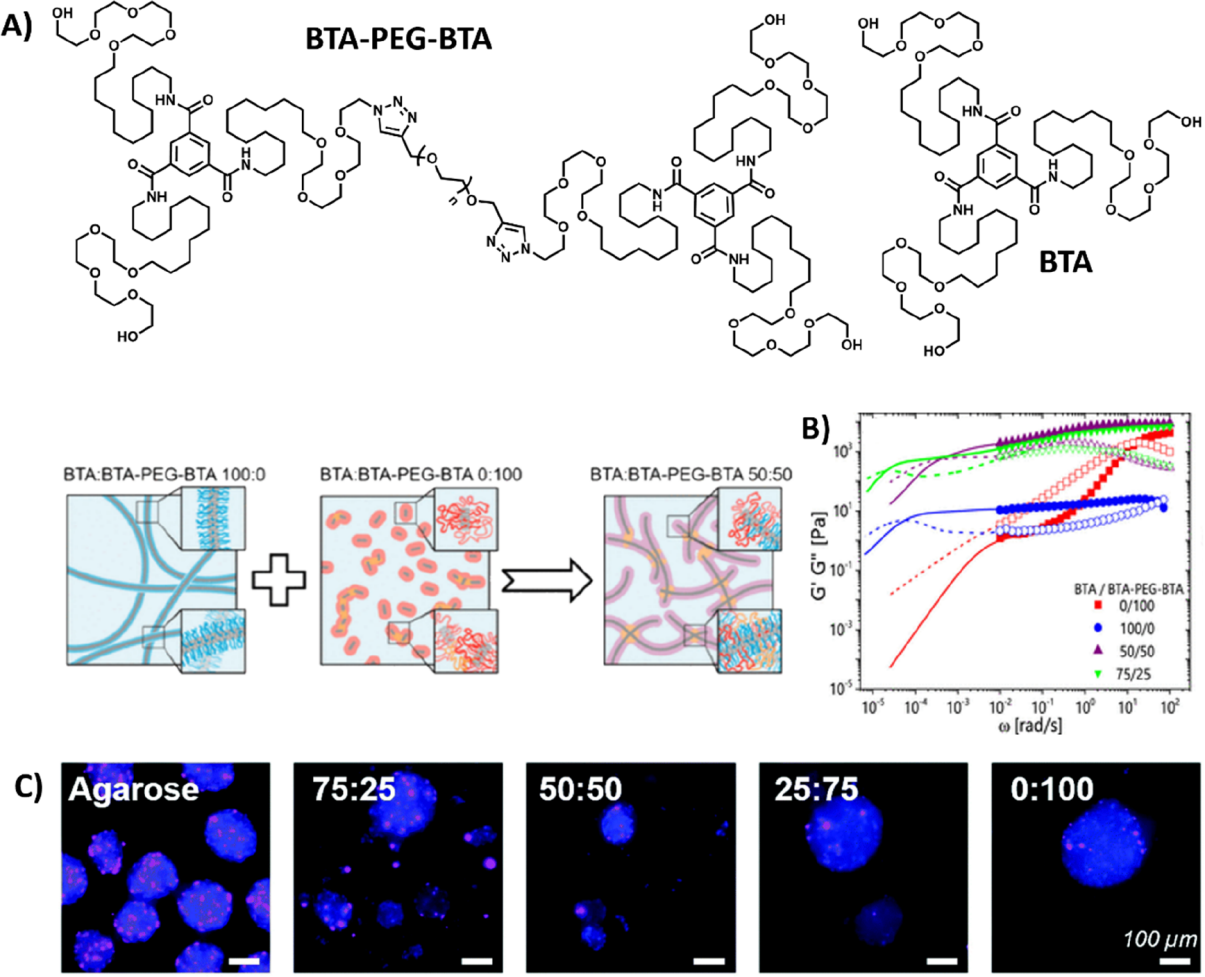
BTA-based supramolecular hydrogel. (A) Chemical structure of BTA, forming long entangled fibers, and a bifunctional BTA–PEG–BTA, forming micelles. Upon mixing, BTA–PEG–BTA can function as a cross-link between BTA fibers. Adapted with permission from ref (110). Copyright 2020 ACS. (B) Mechanical analysis of BTA-based hydrogels, where mixing of the two different BTA molecules results in gels with tunable mechanical properties that are stable over a long range of time scales and show two different modes of relaxation. Adapted with permission from ref (110). Copyright 2020 ACS. (C) Proliferating hMSCs cultured in BTA formulations stained for EdU. Agarose was used as control. Adapted with permission from ref (112). Copyright 2022 RSC.

Another class of supramolecular monomers is *C*_3_-symmetrical discotics, in which the BTA unit has been very well studied by the Meijer lab and more recently by the Baker lab.^102,103^ Translating this supramolecular motif into water, by decorating the core with a C_12_ hydrophobic spacer and a tetra(ethylene glycol) (EG_4_) hydrophilic chain, facilitated the formation of double-helical supramolecular polymers in water (Figure 5A).^37,104^ The formation of fibers is driven by a combination of π–π interactions of the aromatic cores, triple intermolecular amide hydrogen bonding, and hydrophobic effects. By changing the aliphatic chain length, the hydrogen bonding motif becomes more or less shielded from water, respectively, increasing or decreasing the tendency of the monomers to form columnar stacks.^105^ Besides, peptides and carbohydrates can be introduced to the BTA core, revealing the BTA as a versatile platform for biomedical applications.^106−109^ Recently, hydrogels with tunable properties were created by combining two different BTA molecules: a regular BTA mixed with a bifunctional, telechelic BTA–PEG–BTA, i.e., two BTA moieties separated by a PEG spacer (Figure 5A and 5B).^103,110,111^ By creating mixtures between the two BTA molecules with different ratios, different mechanical stiffnesses and dynamics could be achieved, and these differences in physical properties were shown to possess excellent biocompatibility for cellular encapsulation (Figure 5C).^103,112,113^

#### UPy-Based Supramolecular Hydrogels

**Figure 6:**
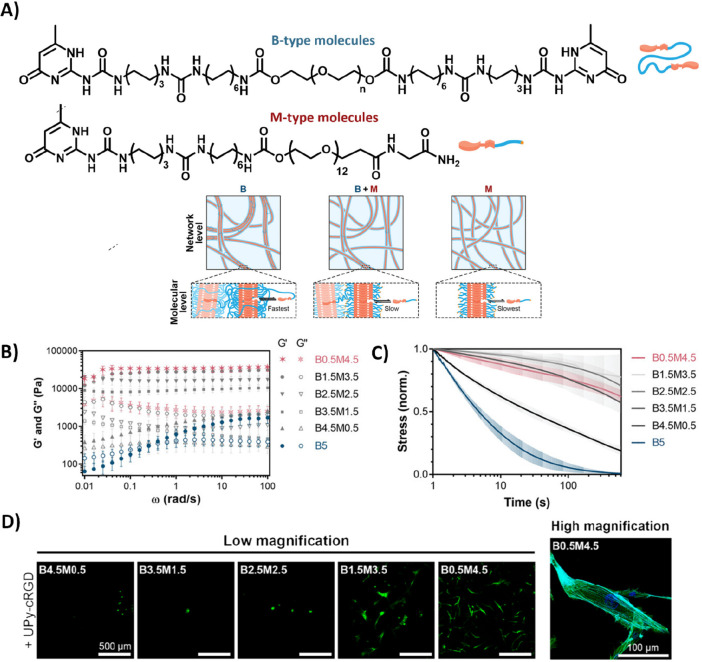
UPy-based supramolecular hydrogel. (A) Different UPy molecules used to form hydrogels, M-type molecules stack into long static 1D fibers and can be functionalized with a large variety of different biochemical end groups. UPy–PEG–UPy forms short dynamic fibers and functions as cross-linkers between M-type molecules. (B) Mechanical properties of UPy-based hydrogels by changing the ratio between B- and M-type molecules; the gel’s mechanical and dynamic properties can be tuned. (C) Changes in dynamics are used to tune cellular adhesion inside the hydrogel. (D) Cell adhesion behavior on UPy hydrogels. Adapted with permission from ref (41). Available under a CC-BY 3.0. Copyright 2021 Wiley-VCH.

UPy molecules form dimers by self-complementary quadruple hydrogen bonding in a DDAA (donor–donor–acceptor–acceptor) fashion, while urea or urethane groups allow for lateral growth (Figure 6A).^114−117^ To allow the assembly of elongated structures in aqueous media, hydrophobic spacers were attached onto the urea groups to shield the hydrogen bonding motifs from water and PEG chains were connected to the hydrophobic linkers as water-compatible units.^118,119^ By tuning the temperature, concentration, or pH, a responsive system^120^ could be created to allow for minimally invasive drug delivery in the heart or the renal organs.^121−123^ Formulation of a fibrillar hydrogel was achieved by introducing a bifunctional UPy–PEG–UPy (Figure 6A).^41^ These molecules act as a cross-linker between the monofunctional UPys to form a fibrous network with variable mechanical and dynamic properties by simply tuning the M/B UPy ratio or changing the hydrogel’s concentration (Figure 6B and 6C). Bioactive UPys could be mixed in the hydrogel as integrin-binding ligands to promote cell adhesion. Cell spreading could be tuned by varying the ratio between M and B UPy (i.e., by changing hydrogel stress–relaxation, Figure 6D).

## Guidelines for Supramolecular Hydrogel–Cell Interactions

**Figure 7:**
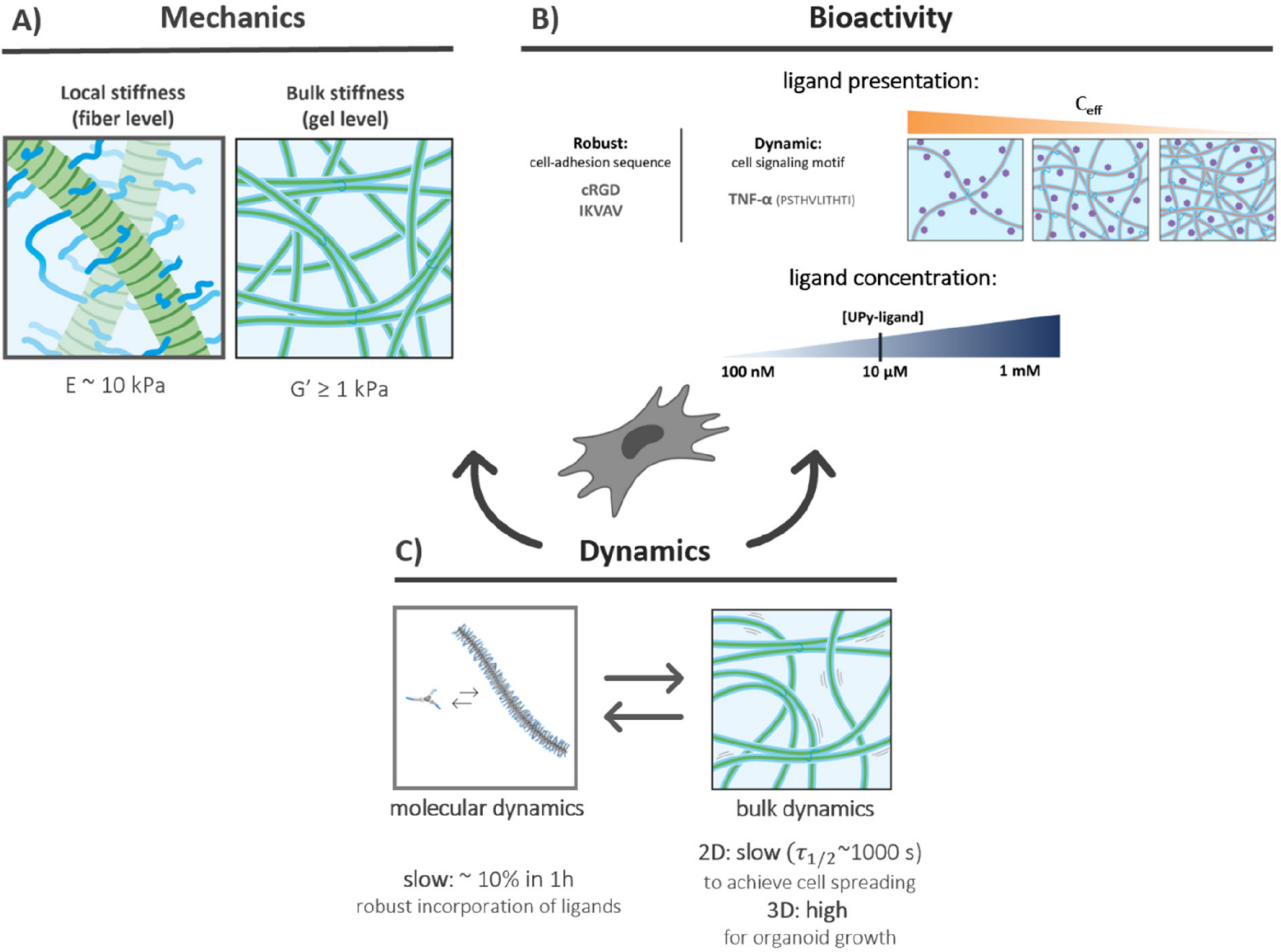
Supramolecular hydrogels as ECM mimics are used to achieve dynamic control over their mechanical and bioactive properties at different length scales (local stiffness, linear stiffness, stress–relaxation, and dynamic or robust presentation of bioactive ligands). Based on the main recent findings, guidelines are proposed for each material property: (A) hydrogel mechanical properties, (B) hydrogel bioactive properties, and (C) hydrogel dynamics to control supramolecular hydrogel–cell interactions. Adapted with permission from refs (113) and (125). Copyright 2023 ACS and Copyright 2023 Wiley-VCH.

By employing the inherent dynamics at the molecular scale (molecular dynamics) and the macroscale (bulk dynamics), control over the hydrogel’s mechanical and bioactive properties is engineered at different length scales, that is, over local stiffness, linear stiffness, stress–relaxation, and the dynamic or robust presentation of bioactive ligands (Figure 7). Based on some recent, important findings, guidelines are proposed to dynamically control cell–material interactions for each matrix property, which are discussed in full detail below.

### Hydrogel Mechanical Properties

Many cell types respond to mechanical cues. Herein, the local and bulk stiffness of hydrogels can be discriminated (Figure 7A). The local stiffness reflects the mechanical properties of a single fiber or a group of clustered fibers at small length scales (approximately nanometers to micrometers), while the bulk stiffness reflects the average stiffness of the whole hydrogel (Figure 7A).^125^ While in most cases the bulk stiffness of the hydrogels is measured and reported, cells might actually “feel” hydrogel stiffness on a much smaller length scale, i.e., on the micrometer or even nanometer level, overlapping with focal adhesion (FA) length scales.^126^ Recent studies indeed confirm this,^126^ with a different number and area of FAs being formed on materials containing different nanometer-scale mechanical properties. The local stiffness of the supramolecular hydrogels was determined by performing nanoindentation on the supramolecular UPy gels using atomic force microscopy (AFM) with a tip of *r* = 750 nm and yielded a Young’s modulus (*E*) of ∼10 kPa. This modulus *E* is calculated from the stress applied and the resulting strain in the linear elastic region of the applied deformation.^127^ Importantly, the local stiffness of the supramolecular hydrogels did not alter upon changing the hydrogel bulk stiffness. However, local inhomogeneities with areas of bundled fibers may still exist within the supramolecular gels, which were indeed confirmed to exist upon further indentation of the sample.^125^ The bulk mechanical properties required for optimal cell performance depend on culture dimensionality. Importantly, the bulk stiffness of the supramolecular hydrogels was determined using shear oscillatory rheology, reporting the storage (*G*′, representing only the elastic response) and loss (*G*′′, representing the viscous component) modulus, in contrast to the Young’s modulus, which was extracted from the indentation experiments. However, these moduli are related to each other by the material’s Poisson’s ratio (ν) through eq 1
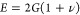
1in which ν ≈ 0.45–0.5 for most hydrogels, yielding *E* ≈ 3 G.

Then, using the bulk stiffness, in 2D, we find that there is a fine balance between stress–relaxation and stiffness; on soft gels (200 Pa), stress–relaxation dictated the cellular behavior. In the case of fast-relaxing gels (τ_1/2_ ≈ 50 s), gels are relaxing cell traction forces, preventing cells from building up tension and hampering cell spreading with that, while on stiffer gels (>1 kPa), the stress–relaxation is submissive to the stiffness. In a 3D matrix, however, cell culture in soft gels (200 Pa) leads to cell aggregates with cell–cell interactions dominating over cell–matrix interactions. When a base level of stiffness (∼1 kPa) is offered to the cells, cell–matrix interactions are dominating with enough mechanical support offered to the cells. Further increasing the stiffness might lead to mechanical constriction, hampering tissue growth for multicellular structures. It is of course well appreciated that in 2D, matrix stiffness affects cell fate, as discovered by Discher lab almost 20 years ago.^128^ In a 3D matrix, however, the story gets more complicated, as increased matrix stiffness often is accompanied by an increased network density and with that of smaller pores. Others found that especially cell migration decreases with matrix stiffness because of more stable FAs as well as due to an increased steric hindrance (i.e., smaller pore size).^129^ Additionally, not all material types allow one to distinguish between effects arising from matrix stiffness or ligand density.

### Bioactive Properties of Hydrogels

For the bioactivity, cells may respond to ligand type, concentration, and presentation, reflected in ligand spacings, valency, patterning, and the effective ligand concentration (Figure 7B).

The type of bioactivity required depends on the cellular aim, which can be broadly divided into (1) cell adhesion sequences and (2) cell signaling motifs (Figure 7B). For both cell types, minimal synthetic sequences as mimics of their natural counterparts can be incorporated into hydrogels. The type of cell adhesion motif and cell signaling sequence need to match with the integrin or cell receptor type, respectively, that are expressed on the cells of interest. The most popular and effective cell adhesion cue is the fibronectin-derived RGD sequence, targeting integrins α5β1 and αVβ3.^130−133^ Variations to the RGD sequence exist, where the cyclic version always outperforms linear versions, owing to its higher conformational resemblance to fibronectin. Other popular synthetic mimics include GFOGER, a collagen-derived sequence, targeting mainly integrin α2β1, and IKVAV,^134,135^ a laminin-derived sequence, which is hypothesized to bind with integrin α6β1 and α6β4.

Often, supraphysiological ligand concentrations (i.e., higher than those used in nature) are used in synthetic hydrogels, including in our supramolecular hydrogels, to achieve a cell response (e.g., cell spreading or correct polarity) (Figure 7B). To illustrate, normally 1 mM of the most effective bioactive UPys (**UPy-cRGD**) is used inside the supramolecular hydrogels. Indeed, diluting these bioactive monomers resulted in loss of cellular spreading or organoid functionality, with the lowest working concentration for **UPy-cRGD** at 10 μM (and being ineffective when *c* ≤ 100 nM). Comparing these synthetic concentrations required for a cell response to the physiological concentrations for their natural counterpart fibronectin, which has a *K*_d_ in the nanomolar regime toward integrin, it indeed shows supraphysiological ligand concentrations are used. These minimal synthetic sequences derived from natural proteins are covalently attached to supramolecular monomers (RGD from fibronectin). Hence, it should be realized that the amino acid sequences surrounding this most active sequence are most likely involved in achieving the optimal orientation to yield the most strong ligand–receptor interactions. This possibly renders these minimum sequences less efficient. An additional explanation for these supraphysiological ligand concentrations includes the availability of ligands in supramolecular systems. As the UPy and BTA supramolecular monomers assemble into bundled fibers and double helices, respectively, a fraction of the bioactive supramolecular monomers might be positioned within these bundles. As a result, these ligands are “shielded”, rendering them less available for binding with cell surface receptors.

Ligand presentation matters. The exact positioning of the bioactive supramolecular monomers within the supramolecular fibers remains unknown (i.e., random distribution driven by entropy or aggregation driven by secondary, intermolecular interactions). Therefore, we are currently engineering geometrical control over ligand presentation into supramolecular hydrogels, reflected in control over ligand spacings and valency with nanometer precision using DNA. In nature, multivalency is key for many cellular processes.^136,137^ A multivalent ligand is composed of multiple ligands (increased valency) as compared to its monovalent counterpart, able to bind to (one or more) receptors with increased affinity. Additionally, multivalency might promote subsequent receptor clustering.^138^ Recently, we introduced the concept of effective ligand concentration (*C*_eff_) in supramolecular hydrogels,^125^ which resulted in multivalent effects in supramolecular systems when *C*_eff_ > 5 mol % bioactive monomer (and when in the correct regime of absolute ligand concentration of from ∼100 μM to 1 mM) (Figure 7B). Control over the effective ligand concentration is realized by (1) keeping the ligand concentration constant but changing the concentration of nonfunctionalized molecules or (2) varying the ligand concentration while keeping the concentration of nonfunctionalized molecules constant (Figure 7). We propose that the higher effective ligand concentration within the supramolecular fibers leads to the multivalency effect as demonstrated by Whitesides and others,^137,139,140^ facilitating cell adhesion.

### Hydrogel Dynamics

Importantly, supramolecular systems display dynamic behavior on different length scales: the molecular dynamics occur on the fiber level (monomer exchange within and between the fibers), while bulk dynamics occur on the gel level (stress–relaxation—fiber rearrangements in the gel, Figure 7C). Concerning the molecular dynamics, the UPy-based supramolecular fibers exhibited barely any monomer exchange (∼10% in 1 h),^113,141^ while the BTA-based supramolecular fibers displayed much faster monomer exchange (∼30–40% in 1 h).^113,141−143^ Importantly, we discovered that the molecular dynamics on the molecular scale is connected and translated to the bulk dynamics on the macroscale for both supramolecular systems: when measured under similar molecular conditions, the UPy gels exhibited slow stress–relaxation (τ_1/2_ ≈ 1000 s) and the BTA gels fast stress–relaxation (τ_1/2_ ≈ 50 s).^113^ With regard to the molecular dynamics influencing the cellular behavior, we observed cell spreading on and in the robust UPy supramolecular system, while only cells with a round morphology were found for the more dynamic BTA supramolecular system. These findings show that the robust incorporation of cell adhesion ligands is crucial to achieve cell spreading; otherwise, the cells will pull out the bioactive monomers from the stack (Figure 7C). To compare our findings regarding the molecular dynamics influencing the cellular behavior to others, the Stupp lab recently also highlighted that greater supramolecular motion (using both homo- and coassemblies) of their bioactive peptide amphiphiles (PAs) promoted spinal cord injury recovery. They hypothesized that the greater motion facilitated receptor clustering by being able to orientate toward receptors regardless of PA orientation, eventually resulting in effective signaling.^90−92^ Taking this together, we propose that to achieve cell adhesion, cell adhesion cues must be robustly incorporated inside supramolecular fibers, while cell signaling cues (such as mimics of growth factors) should be dynamically delivered to cells with greater supramolecular motion to allow receptor–ligand interaction regardless of the ligand orientation, resulting in efficient cell signaling.^90,92^

The bulk dynamics (stress–relaxation) required to achieve cell spreading and tissue growth is heavily dependent on the culture dimensionality (2D versus 3D). In 2D, we found that on soft gels (∼200 Pa), slow stress–relaxation (τ_1/2_ ≈ 1000 s) is crucial to achieve cell spreading (Figure 7C). In contrast, cells cultured on fast-relaxing gels (τ_1/2_ ≈ 50 s) remained round in morphology. We hypothesize that the quick fiber rearrangements relaxed cell traction forces, hampering cell spreading. However, on stiffer gels (∼1 kPa), gel stiffness overruled gel dynamics, with cell spreading on the fast-relaxing gels as well. Additionally, the results above were dependent on cell type, with fibroblasts clearly responding “faster” than epithelial cells. To put these results in perspective on how the material stress–relaxation affects cell spreading, the Venoy, Burdick, and Chaudhuri laboratories showed through experiment and modeling that indeed the material viscoelasticity (but influenced by material stiffness) is the regulator of cell spreading. This was demonstrated by comparing substrate relaxation time scales with that of clutch binding (∼1 s) and subsequent FA lifetimes. FA lifetime varied between ∼10 and 100 s but increased toward minutes upon increased material stiffness. Consequently, they found that cell spreading is impaired when substrate relaxation occurred faster than clutch binding and FA lifetime, while for stiffer substrates, viscosity did not influence cell spreading anymore, as the molecular clutches were already saturated by the elevated stiffness which resulted in longer FA lifetimes.^144^ The relaxation time scale of the supramolecular hydrogels spans from ∼30 s toward 1000 s, overlapping with cellular time scales and agreeing with the above findings.

Moving toward 3D culture, it should be realized that slow-relaxing gels might cause mechanical constriction, hampering cell spreading and tissue growth. On the contrary, fast-relaxing gels could dissipate tissue forces through stress–relaxation mechanics, promoting cell migration and growth, which is desired (Figure 7C).

The Chaudhuri, Mooney, Garcia, and Anseth laboratories also revealed the crucial importance of matrix dynamics in 3D utilizing other material types. To achieve cellular spreading, the Chaudhuri lab showed that their intrinsically dynamic, hybrid alginate–PEG gels with fast relaxation (fast, τ_1/2_ ≈ 60 s; slow, τ_1/2_ ≈ 1 h) facilitated cell spreading, proliferation, and differentiation.^145^ Similar results were found by the Anseth lab, utilizing dynamic covalent, boronate-based gels with relaxation times of seconds or less, showing their fast-relaxing matrices favored cell–matrix interactions with cellular spreading and YAP nuclear translocation.^146^ To enable cyst formation, the Garcia lab showed that ECM degradation by proteases (external dynamics) was required in their PEG-based synthetic covalent gels.^147^ The Mooney lab showed that fast stress-relaxing hybrid alginate–PEG gels (fast, τ_1/2_ ≈ 30 s; slow, τ_1/2_ ≈ 350 s) even promoted tissue growth dynamics, owing to cells being able to remodel their matrix.^148^ We have shown using supramolecular systems that the dynamics of the 3D matrix can directly impact and control the formation of cellular spheroids,^149^ while higher dynamics in dynamic covalent systems can lead to better maturation and morphology of encapsulated kidney organoids.^150^

## Guidelines and Conclusions

Along this perspective, we briefly recap how nature uses only a limited number of molecules which can assemble into a large library of materials with different stiffnesses, dynamics, bioactive properties, and special processes such as stress–relaxation. Current synthetic materials often struggle to recreate the numerous properties of the ECM. Often, a material is great to represent one specific property of the ECM; for other properties, a completely different material is needed. Supramolecular hydrogels are arising as the next-generation biomaterials to their modularity and inherent dynamics, allowing for dynamic control over complex mechanical and bioactive features across different length scales. Like in the natural ECM, these systems can assemble into larger, hierarchical complexes with tunable mechanical, dynamic, and bioactive properties. Based on some recent, important findings, guidelines underlying supramolecular hydrogel–cell interactions are proposed (Figure 7).

- Regarding (1) gel mechanics, a base level of bulk stiffness (*G*′ ≈ 1 kPa) is required to offer mechanical support to cells, especially in 3D, to achieve cell adhesion.^113,125^ However, stiffer gels (with inherent increased network density and smaller pores) might lead to mechanical constriction, hampering tissue growth. However, soft hydrogels will fulfill the needs of cells that do not rely on cell–matrix interactions as much.
- Lessons learned for (2) the bioactivity are (i) the ligand type needs to match with the expression of the receptor of interest,^41,125^ (ii) supraphysiological ligand concentrations are required in synthetic gels^41,125^ (most likely due to steric hindrance/inefficient ligand orientation toward receptors), and (iii) when *C*_eff_ ≥ 5 mol %,^125^ multivalent effects can be observed through facilitating ligand recruitment.
- For (3) the gel dynamics, slow molecular dynamics (∼10% in 1 h) are required to achieve cell adhesion to withstand cell-pulling forces.^113^ The bulk dynamics is heavily dependent on culture dimensionality: in 2D, slow stress–relaxation (τ_1/2_ ≈ 1000 s) is crucial to achieve cell spreading and prevent the relaxation of cell traction forces, while in 3D, fast-relaxing gels (τ_1/2_ ≈ 50 s) promote the growth of single cells into multicellular organoids by dissipating tissue forces.

Importantly, different cell types require different environments to achieve optimal cell performance. Here, culture dimensionality matters (2D versus 3D culture) as well as cell type (single cells versus multicellular organoids). So, design criteria for the biomaterial to achieve optimal cell performance differ and should be tailored toward cell type. The supramolecular toolbox allows one to tailor materials properties toward the precise needs of a cell or a specific ECM.

## Limitations and Considerations

Despite recent great advancements of supramolecular systems, certain limitations and considerations persist. These include the importance of formulation, the introduction of complex mechanical features, control over bioactive ligand presentation, the conformation and complexity/type, as well as the delicate role of time scales, which are discussed below.

(1) Control over the hierarchical structure is key to performance. Collagen is an excellent example of a natural biopolymer with different properties based on its bioassembly pathway. From strong, persistent collagen I to the soft, transient collagen IV, a singular biopolymer can be given diverse function based on differences in assembly and post-translational modifications. While traditional covalent hydrogels often have hierarchical information imparted via processing steps, supramolecular hydrogels offer unique opportunities for providing diverse morphologies based on formulation and processing conditions.^54,151^ Though the potential polymorphism during self-assembly of supramolecular assemblies can be a challenge,^52,54,84^ gaining control and direction over this process has the potential to offer emergent properties from singular systems with high reproducibility rates,^152−155^ as also recently highlighted by Adams et al.^155^ While a difficult area of research, new analytical tools and methodologies, like intermediate quality control steps, set this up to be a promising area of innovative structure–property relationships in the near future.
(2) Mechanoresponsiveness and complex, dynamic mechanical properties are abundant and important in natural systems.^156,157^ In nature, for example, the Ruberti and Dunn groups have shown that the enzymatic degradation load of collagen is dependent on the applied mechanical force.^158,159^ Likewise, fibrins’ bioactivity is regulated through a mechanochemical feedback loop; when fibrin is under mechanical stress, decreased binding of fibrin and platelets was observed, yielding less activated platelets.^160^ Hence, of particular interest in the future is the combination of supramolecular interactions with mechanoresponsive elements, such as Förster resonance energy transfer (FRET) sensors,^161^ as many ECM functions are regulated by cellular tension.
Additionally, the introduction of complex mechanical features, like stress stiffening as observed in fibrin,^162^ remains challenging in synthetic supramolecular systems. To date, few examples exist, including the successful introduction of stress stiffening in bis-urea supramolecular polymers. Herein, the stress stiffening was hypothesized to occur due to soft bending modes present in bundled bis-urea fibers.^42^ Additionally, dynamic covalent strategies have been elegantly employed to introduce such stiffening features in synthetic hydrogels, which could be tuned as function of polymer concentration, pH, and temperature,^163^ where a combined entropic and enthalpic elasticity is hypothesized to cause these stress-stiffening features.
(3) Supraphysiological ligand concentrations (i.e., higher concentrations than those used in nature) are required in synthetic supramolecular hydrogels to achieve a desired cell response, like cell spreading. Often, minimal synthetic sequences derived from natural proteins are covalently attached to supramolecular monomers (e.g., RGD derived from fibronectin). Such minimal synthetic sequences contain a lower *K*_d_ as compared to natural ligands,^164^ which could explain why supraphysiological ligand concentrations are required in synthetic hydrogels to achieve a biological response. Additionally, part of the bioactive, supramolecular monomers (i.e., the ligands) might be “shielded” inside the bundled supramolecular fibers, lowering their availability toward biological entities. To overcome and better understand (the need for) supraphysiological ligand concentrations, future research may focus on strategies to present ligands in a preorganized fashion using DNA^165^ versus dynamically.^90,113^
(4) The local folding within natural biopolymers gives rise to its secondary structure, which is key to achieve bioactivity. The most abundant secondary structures of proteins are alpha helices and beta sheets. Many synthetic ligands are minimalistic versions of their natural counterparts (i.e., IKVAV derived from laminin^134^) and therefore lack crucial amino acids surrounding this active sequence which are required to achieve the desired, correct confirmation. Recapitulating this secondary structure and introducing control over folding in synthetic hydrogels will be essential to accurately mimic the bioactivity found in the biological ECM. Recent work by the Rosales lab recognized this and showed that the hydrogel mechanical properties could be tuned by controlling the peptoid sequence and structure.^166^
(5) To arrive at a greater level of biological complexity, multiple short, bioactive synthetic sequences could be mixed together. Integrin expression on cells is often a mix of different subunits, resulting in the need for different bioactive sequences. Often only one minimalistic sequence is included as the ligand in synthetic gels, while nature uses a complex interplay of different proteins (e.g., fibronectin) and carbohydrates (e.g., glycosaminoglycans), which synergistically catalyze cell adhesion and focal adhesion formation.^167−169^ Future work may focus on introducing larger peptides, whole proteins, and carbohydrates onto supramolecular fibers. This will be a challenging endeavor and interesting area of research as bulky and large groups might interfere with the stability of the whole supramolecular fiber.
(6) The ability to engineer nondegradative dynamic time scales into materials is very appealing and interesting and allows probing of fundamental cell–material time scales. However, these time scale-dependent properties^170,171^ require a delicate balance between dynamics and persistence. Stress–relaxation has come to the forefront as one such time-dependent process, with precise control afforded over the tuning of dynamic cross-links and assemblies in numerous systems,^58,146,172−175^ showing key importance of the cellular morphology and ultimately function. However, while stress–relaxation is a bulk phenomenon, the molecular dynamics of individual assemblies has also come into light as having discernible effects.^90,176^ Furthermore, the kinetics and dynamics of cell adhesion can be probed by careful control over supramolecular assemblies^41^ and is an active area of study.^155,177,178^ Moving from a bulk materials view on cell–matrix interactions toward a more molecular view (especially in view of multicomponent systems) will undoubtedly lead to more advanced hydrogel systems with emergent behavior.

Here, we would also like to add that the proposed design principles are just guidelines. This is what we have observed over many studies in the field yet are invited to be tested and understood in different contexts. Refining these guidelines, adding to them, and challenging them is best done via a community effort facilitated by open discourse. We may never arrive at a “Lipinski rule of 5”,^179^ as in drug discovery (though this has long been challenged). Yet, even the attempt can further the field. Lastly, the guidelines for cell–matrix interactions in supramolecular materials is only a piece in the puzzle. As we create novel breakthroughs in the creation of cell–material constructs, characteristics like processability, tissue formation, cell maturation, cell migration, and others become critical. Trends for these characteristics are also rigorously under discovery and may be of benefit from defined guidelines as well.

## Future Prospects

While chemists and material scientists are making significant efforts to create advanced functional supramolecular hydrogels that mimic key features from the native ECM, here we propose several important prospects to be considered when designing future more life-like ECM-mimicking materials. Besides the importance of engineering materials (proposed materials aspects), the field of synthetic biology has emerged to engineer and manipulate the cell (proposed cellular aspects).

### Material Aspects

**Figure 8:**
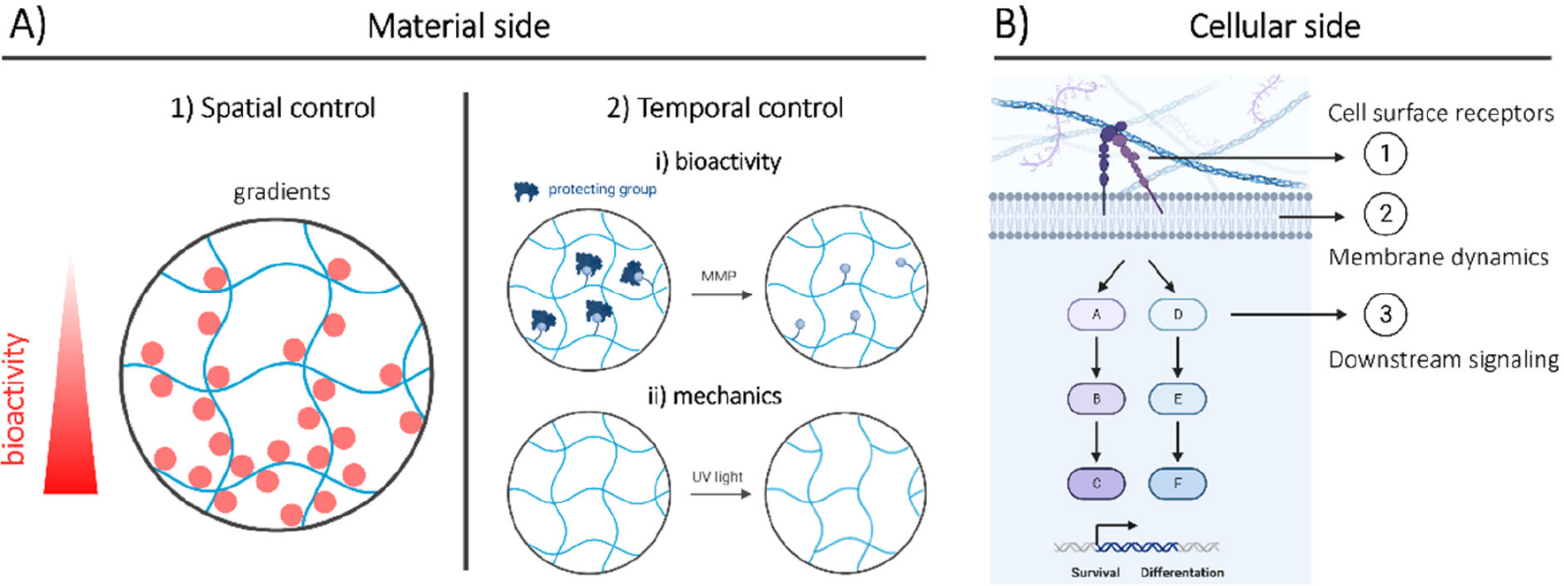
Implications for developments on the material and cellular side. (A) Material side: engineering spatiotemporal control over hydrogel properties. (1) Spatial control allows for ligand gradients and/or high local control over ligand concentrations (e.g., through microgels). (2) Temporal control allows one to control (i) gel bioactive and/or (ii) mechanical properties in time through the smart use of stimuli-responsive groups, like enzymes (e.g., matrix metalloprotease (MMP) sensitive) or light-responsive ones (e.g., *o-*nitrobenzyl groups). (B) Cellular side: manipulations to increase understanding of cell–material interactions through manipulations of (1) cell surface receptors, (2) membrane dynamics, and (3) downstream signaling. Figure created using Biorender software.

(1) Combining covalent bonds with dynamic self-assembly will be key. Collagen is a wonderful example of a supramolecular self-assembled material; it provides strength and maintains remodeling in many tissues. Yet, the performance of collagen does not rely solely on self-assembly. Important cross-linking after assembly (i.e., aldol condensation) is crucial. In numerous biological assemblies (e.g., titin, elastin, fibrin) we can observe the powerful effect of covalent and supramolecular interactions. Work has started in the community on the combinations of these two bonding types in advanced supramolecular structures,^42,111,180−185^ consistently resulting in impressive properties when a proper balance is found. Recent results from our laboratories show that covalent reinforcement of supramolecular fibers can be done in the presence of cells without greatly affecting the shear modulus of the material.^186^ Expanding this research to include more biomimetic approaches to control the dynamics and self-assembled structure has the potential to lead to responsive structures with impressive properties in the near future.
(2) Future advancements may focus on engineering spatiotemporal control over hydrogel properties, i.e., in space and in time. To achieve spatial control (1), gradients of bioactive signals might be introduced into hydrogels or local control over ligand concentrations can be introduced (Figure 8A-1). Microgels can be used to achieve such gel properties, allowing a high local control—as demonstrated by, for example, the Segura, Anseth, and de Laporte laboratories.^187−191^ To achieve temporal control (2), stimuli-responsive groups, such as light- or enzyme-sensitive ones, can be incorporated into the polymeric backbone to change the gel mechanical or bioactive properties over time (Figure 8A-2).^156,192,193^ For example, certain bioactive signals that are crucial during early stages of cellular growth can be cleaved during later stages of culture (Figure 8A-2i). Likewise, a cleavable group could allow switching from a stiff hydrogel toward a softer hydrogel over time, which could be advantageous for culturing multicellular structures, which will exert more forces on the material over time (Figure 8A-2ii).
(3) A screening approach combined with artificial intelligence will be key to processing and assessing the performance of a large library of different material combinations on cellular outcome. Joining forces through experiment and modeling will be very important.^194−196^ The field has made some initial steps toward this direction, where several groups, like the Weil lab, have started to utilize data-mining strategies,^197^ Bayesian optimization,^198^ or a design of experiments approach^199^ to study intermediate-sized data sets.
(4) While the native ECM constantly undergoes energy-intensive remodeling and responds to cellular cues, engineered matrices exhibits rudimentary out-of-equilibrium behavior^200,201^ and cell communication.^202^ Out-of-equilibrium information processing and signal transduction within synthetically designed hydrogels remains a significant challenge. Combining cell-responsive elements with out-of-equilibrium assemblies into truly dissipative systems, as pioneered by the Hermans, Whitesides, Boekhoven, Eelkema, van Esch, and Konkolewicz laboratories,^192,203−208^ is an attractive area of progress in the near future.

### Cellular Aspects

(1) We can leverage biology. Recent advances in synthetic biology have made it possible to create designed biomaterials using cells as the chemical factory on a small scale. Synthetic chemistry has enabled the rational design of biomaterials, able to influence cells on a larger scale. Although these two fields have evolved separately, there are several calls to merge progress,^209−213^ including a very recent report by DeForest and Anseth et al.,^213^ toward the creation of living materials.^214,215^ Here, one can envision the chemical design of a scalable hydrogel, which can be manipulated by a cell. Recent collaborative work by Kietz and Rosales lab shows this is possible.^216,217^ Further innovations can leverage the synthetic material to create the environment and 3D shape, while the living cells provide the biological complexity and production of biological molecules. Using living units as sensors and processors to create stimuli-responsive, shape-changing, and adaptive materials could herald new advances for major challenges in biomedicine and sustainability. Only by collaboration and familiarization between these two fields will we be able to unlock the potential of chemical synthesis and chemical biology for advanced materials.
(2) Manipulations on the cellular side will result in useful information underlying the cell–material interaction with respect to surface receptors participating in it, ligand recruitment in combination with receptor clustering, and the subsequent underlying mechanotransduction pathways involved (Figure 8B). (1) Cell surface receptors can be inhibited or enhanced using, for example, integrin activating or inhibiting antibodies.^125^ Additionally, (2) cell membrane mobility can be manipulated to investigate to what extent the clustering of cell surface receptors (focal adhesion formation) are involved in cell adhesion response. To achieve this, membrane fluidity could be modulated using membrane “softening” or “stiffening” drugs, like methyl-beta-cyclodextrin (MCBD)^218^ or glycerol,^219^ respectively. With regard to (3) downstream signaling, manipulating the cellular contractile system through Rho-kinase (ROCK) inhibitors could elegantly be used to investigate how the cellular contractile system is involved in the cell–material interaction.^125^

Taking the manipulation on the cellular side one step further, cells can be genetically modified such that they can specifically interact with a target of interest (i.e., specific functional materials or specific cell types such as cancer cells), regardless of their regular expression of cell surface receptors. One example of such genetic manipulation is genetically targeted chemical assembly (GTCA), as recently developed by the Bao and Deisseroth laboratories, which allows the in situ attachment of functional materials on specific genetically modified cells (e.g., streptavidin).^211^ Alternatively, receptor expression could be genetically modulated, through (1) the expression of synthetic receptors (e.g., chimeric antigen receptor (CAR) T-cell therapy to reprogram T cells)^220,221^ or (2) tuning receptor expression^222^ for improved control and precision over immunotherapy.^223,224^

### Toward Innovative Applications

**Figure 9:**
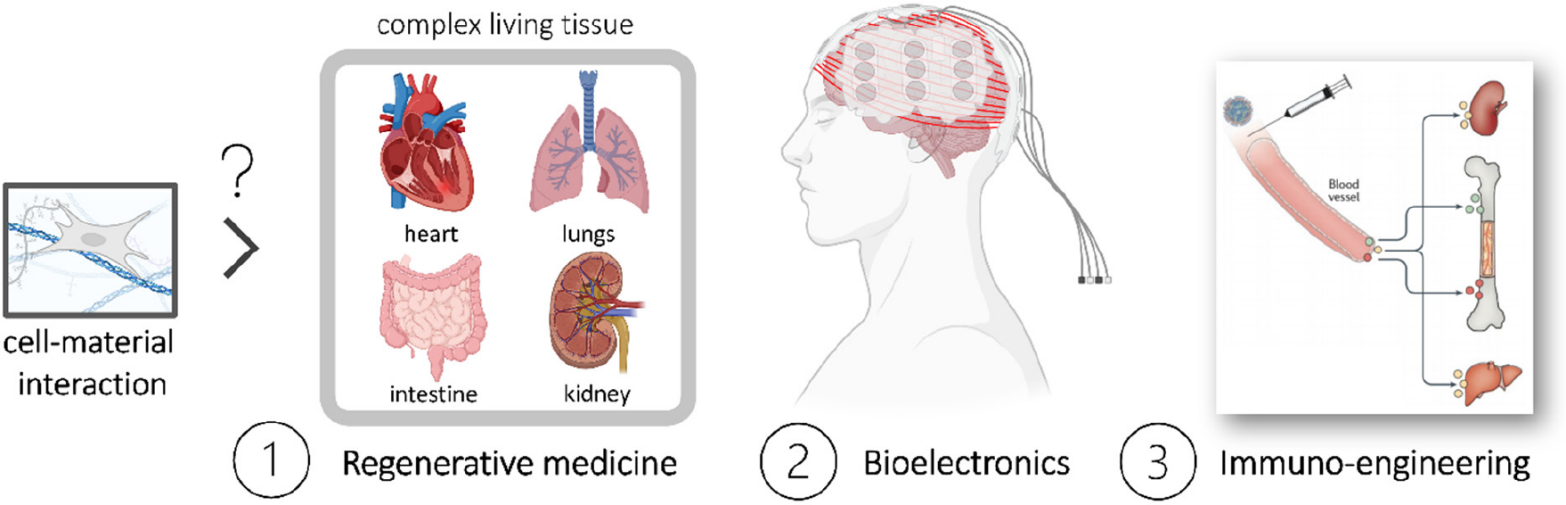
Future work may utilize these fundamental guidelines underlying the cell–material interaction to advance fields like regenerative medicine (1), bioelectronics (2), and immunoengineering (3), opening new possibilities for innovative applications. Figure created using Biorender software.

Future work may utilize these guidelines underlying the cell–material interaction to advance not only the regenerative medicine field (1) but also emerging fields like bioelectronics (2) and immunoengineering (3) (Figure 9), opening new possibilities for innovative applications. With regard to regenerative medicine (1), these fundamental guidelines underlying the cell–material interaction may be utilized to culture more complex living tissue in a fully controlled, synthetic fashion. These complex in vitro cultures hold promising applications in drug screening or as tissue replacements.^225^

Bioelectronics (2) are very promising for monitoring physiological function and restoring diseased body functions.^4^ Today’s bioelectronics show already matching stiffness with biological tissue and dynamic behavior, enabling adaptability to body movements.^1,3,5,226,227^ Challenges here, however, are found in retention times and cellular specificity after implantation in living tissue. The obtained cell–material rules from our work might be implied here to improve current state-of-the-art conductive materials with improved retention and cell-type specificity.

Lastly, immunoengineering (3) is a relatively new field that manipulates the immune system with applications in oncology, transplantation, and infectious disease.^228,229^ As most immune cell therapies are administered systematically, they suffer from low efficiency and low targeting precision. Biomaterial strategies can facilitate local immunomodulation.^230^ Importantly, also this regulation is founded by control over cell–material dynamics.
